# Use of recombinant *Brucella* outer membrane proteins 19, 25, and 31 for serodiagnosis of bovine brucellosis

**DOI:** 10.14202/vetworld.2020.1439-1447

**Published:** 2020-07-25

**Authors:** Aitbay Bulashev, Orken Akibekov, Alfiya Syzdykova, Zhanbolat Suranshiyev, Bakytkali Ingirbay

**Affiliations:** Department of Microbiology and Biotechnology, Faculty of Veterinary and Livestock Technology, S. Seifullin Kazakh Agrotechnical University, Nur-Sultan, Kazakhstan

**Keywords:** *Brucella*, diagnosis, enzyme-linked immunosorbent assay, outer membrane proteins

## Abstract

**Background and Aim:**

Brucellosis remains one of the most common zoonoses. The current anti-brucellosis measures are largely deemed ineffective due to a lack of specificity of conventional serological tests. This study evaluated the use of *Brucella* outer membrane protein (Omp)19 for serodiagnostic testing.

**Materials and Methods:**

The antigenicity of recombinant *Brucella* Omp19, Omp25, and Omp31 was examined in serum samples from mice and rabbits immunized with Omp19 or *Brucella abortus* 19 whole cell (WC) and 12 and 152 cows experimentally or naturally infected with brucellosis, respectively. Serum samples were collected from 151 cows that were vaccinated with *B. abortus* 19 and 12 unvaccinated heifers that were maintained on a brucellosis-free farm.

**Results:**

Immunization with Omp19 resulted in antibody production in mice after a single injection without the use of adjuvant. Serum antibodies obtained from rabbits immunized with inactivated *B. abortus* strain 19 WC targeted Omps by enzyme-linked immunosorbent assay (ELISA) and Western blot. Antibodies targeting Omp19 were identified in all *B. abortus* strain 544 experimentally infected cows at day 14 post-inoculation (p.i.); Omp25 was detected by ELISA at day 28 p.i., while an ELISA targeting Omp31 was negative for 25% of cows at this time point. Omp19 and Omp25 were readily detected by sera from cows from a new epizootic focus. Antibodies recognizing Omps were also detected in >50% of the animals maintained in a brucellosis-free herd at 10 months after vaccination.

**Conclusion:**

*Brucella* Omp19 in combination with Omp25 and Omp31 may be utilized as target antigens in an ELISA designed for serological testing of unvaccinated cattle.

## Introduction

Brucellosis has been identified as a zoonosis that results in significant reductions in livestock productivity, which, in turn, poses a serious threat to human health worldwide. The disease involves considerable health-care costs and reduces animal productivity; therefore, these factors are of significant concern in regions that rely heavily on current economic growth in an effort to reduce poverty [1]. Despite some progress in managing brucellosis, epidemic situations remain complicated in Asia, Africa, Latin America, the Middle East, as well as in the Mediterranean and Southeast regions of Europe [2].

Brucellosis is currently endemic in countries within Central Asia, including the Republic of Kazakhstan (RK) [3]. One of the main reasons for the low efficiency of anti-brucellosis measures is the lack of specificity and sensitivity of serological tests used in the diagnosis of the disease. Conventional serological tests such as the rose Bengal test (RBT), the agglutination test (AT), and the complement fixation test (CFT) are used for diagnosing bovine brucellosis in Kazakhstan. The main target antigen of these tests is the lipopolysaccharide (LPS) of smooth *Brucella* strains (S-LPS), which are known to be involved in the cross-reactions with closely related bacteria, for example, *Yersinia enterocolitica* and *Escherichia coli* [4]. Moreover, the use of antibodies targeting S-LPS precludes differentiation of *Brucella*-infected animals from those vaccinated with attenuated smooth strains of these bacteria [5]. Protein antigens, including those shielded by LPS, are currently the focus of attention among those involved in the development of brucellosis vaccines and diagnostic agents [6].

Recombinant DNA technology has paved the way for new prospects with respect to the use of individual proteins in diagnostic and/or prophylactic preparations. The use of recombinant *Brucella* proteins with constant immunogenic and antigenic properties generated from harmless producer strains would improve standardization compared to methods in current use that feature complex antigenic preparations obtained using traditional technologies [7]. Among the non-polysaccharide components of *Brucella* spp., the outer membrane proteins (Omps) are of significant interest in this regard. Omps are divided into three groups according to their apparent molecular weights (MWs); among these are Group 1 at 88-94 kDa, Group 2 at 36-38 kDa, and Group 3 at 31-34 and 25-27 kDa [8]. In addition, *Brucella* proteins with MWs of 10, 16, and 19 kDa have also been described [9]; these have been identified as lipoproteins [10]. Earlier studies have suggested the use of native *Brucella* proteins [11] and non-LPS antigens [12] for serological differentiation of infected from vaccinated cattle. Subsequently, recombinant proteins, including rOmp10, rOmp16, rOmp19, rOmp25 and rOmp36 [13], rOmp31 [14], rOmp28 [15,16], alone, or in combination (rOmp10 + rOmp19 + rOmp28) [17], were also tested to detect *Brucella*-specific antibodies in ruminants sera. The potential of rOmps with respect to the serological diagnosis of brucellosis remains to be poorly understood, and the results obtained were sometimes contradictory. In a mouse model, indirect enzyme-linked immunosorbent assay (ELISA) based on a combination of three Omps (Omp25 + Omp28 + Omp31) was able to differentiate antibodies developed in response to *Brucella melitensis* from those associated with vaccines or that were non-specific and cross-reactive [18]. However, our previous study revealed that serum antibodies directed against Omp25 and Omp31 could be detected not only in seropositive unvaccinated cows but also in some healthy animals vaccinated against *Brucella abortus* strain 19 [19]. The antigenicity of Omp19, and the possibility of using this protein to differentiate between these two conditions, remains to be unexplored. Omp19 is currently identified as one of the most promising components with respect to the creation of new vaccine preparations; as such, its potential for cellular induction rather than humoral immunity is still under study [20]. However, as noted by Verma *et al*. [21], any study of immunity against brucellosis will also need to include a humoral approach rather than focusing solely on cell-mediated immunity.

The aim of this study was to examine the use of recombinant *Brucella* Omp 19, 25, and 31 for serodiagnosis of bovine brucellosis

## Materials and Methods

### Ethical approval

All procedures involving animal care were performed in accordance with the Guidelines for Accommodation and Care of Animals: Species-specific provisions for laboratory rodents and rabbits (Interstate Standard, GOST 33216-2014). Care and Use of Laboratory Animals was approved by the Animal Ethics Committee, Faculty of Veterinary and Livestock Technology, S. Seifullin Kazakh Agrotechnical University (KATU), Nur-Sultan, Kazakhstan. All blood samples from cattle were taken by well-trained veterinarians with respect to animal welfare using a closed blood sampling system (JSC “ZTOWN Development,” Kazakhstan) and transported to the laboratory immediately. After centrifugation, the separated serum was poured into sterile tubes and stored for up to 24 h in a refrigerator at +4°C, but for longer storage, it was aliquoted and stored frozen at −20°C until used.

### Animals

The study was conducted from January to March 2019. Eighteen outbred male mice (9-10 weeks, 20-25 g body weight) and two male Soviet chinchilla rabbits (6 months, 3300-3500 g body weight) were maintained under hygienic conditions in the vivarium of KATU. The animals were provided with food and water *ad libitum*. A 12 h light-dark cycle was maintained in the animal housing and the temperature (22±2°C) and humidity (55-60%) were monitored daily.

### Preparation of B. *abortus* 19 whole-cell (WC) suspension

*Brucella* cells were cultured on erythritol agar (Microgen, Makhachkala, Russia). The culture was examined for typical growth and purity, then washed with solutions containing 0.5% phenol in phosphate-buffered saline (PBS), and kept in an incubator at 37°C for 48 h to inactivate the bacterial cells. A cell suspension was prepared and washed 3 times by centrifugation at 5000 rpm for 30 min.

### Recombinant proteins

The recombinant *Brucella* proteins described in our previous studies and used here were rOmp19 [22], rOmp25, and rOmp31 [23]. HisTrap columns (GE Healthcare Life Sciences, Cardiff, UK) were used to purify recombinant proteins as per the manufacturer’s instructions. The properties of the target proteins were confirmed by Western blot using anti-His Tag monoclonal antibodies conjugated with horseradish peroxidase (HRP, Thermo Fisher Scientific, Waltham, USA).

### Immunization of mice with *Brucella* recombinant (r) Omp19

To determine the capacity for antibody production, this study featured six groups of mice, with three mice per group. Mice in Group I were immunized once through the subcutaneous (s.c.) route with Freund’s complete adjuvant (FCA) alone, and those in Groups II, III, and V were immunized once with rOmp19 in PBS (pH 7.2–7.4), with Freund’s incomplete adjuvant (FIA) or with FCA (Sigma-Aldrich, St. Louis, USA), respectively. Mice in Group IV were immunized twice with rOmp19 mixed with FIA and with PBS on days 0 and 14, respectively. Group VI was immunized thrice according to the following scheme: rOmp19 + FCA, rOmp19 + FIA, and rOmp19 + PBS on days 0, 14, and 21, respectively. Each inoculation included 25 μg of the recombinant protein. Blood was sampled from the tail vein on day 21 (Groups I, II, III, and V) and day 28 (Groups IV and VI); sera isolated after centrifugation were used to determine antibody titers against rOmp19, rOmp25, and rOmp31 by ELISA. Negative control sera were obtained from mice on day 0 before the first immunization.

### Determination of the anti-rOmp19 titer in murine antisera

Wells in polystyrene plates (Thermo Fisher Scientific, Waltham, MA, USA) were coated with rOmp19, rOmp25, or rOmp31 at 5.0 μg/mL in bicarbonate buffer, pH 9.6 and incubated at 4°C overnight. The plate was then washed 3 times sequentially with PBS and PBS supplemented with Tween-20 (PBS-T). Antisera dilutions were prepared in PBS-T (1:100–1:12,800) which were added to 8 wells coated with each of the proteins; the plate was incubated at 37°C for 1 h. After washing, (HRP)-conjugated rabbit anti-mouse IgG (Sigma-Aldrich, St. Louis, MO, USA) diluted with PBS-T was added to each well. The plate was incubated for another 1 h at 37°C, washed 3 times, and then developed with O-phenylenediamine (Sigma-Aldrich, St. Louis, USA). The reactions were stopped after 3-5 min by adding an equal amount of 2 M sulfuric acid to each well. The absorbance was measured at 490 nm using a plate reader (Bio-Rad 680, Redmond, WA, USA). The cutoff value for the assay was calculated as the mean optical density (OD) plus 3 standard deviations for 18 negative control sera obtained from mice before immunization.

### Production of anti-*B. abortus* 19 WC antibodies

Rabbits were immunized s.c. with a suspension of phenol-killed cells as previously described [18]. Blood was sampled from the marginal vein on day 0 as negative controls with each rabbit under sedation; blood was sampled again on days 14, 21, 35, and 45 to evaluate the antibody response. The serum samples were stored at −20°C until use.

### Collection of serum samples

A total of 327 cattle sera were evaluated. Of these, 12 serum samples were from cows that underwent an experimental infection with the virulent strain *B. abortus* 544; this was kindly provided by Professor K. Tabynov, Head of the Laboratory for the Prevention of Infectious Diseases, Research Institute for Biological Safety Problems, RK. Serum samples from 152 seropositive unvaccinated cows identified from a fresh epizootic focus of brucellosis infection were obtained from the Collection of National Reference Center for Veterinary Medicine, Ministry of Agriculture, RK. A total of 151 serum samples were obtained from revaccinated cows housed at the Mereke farm (Bukharzhyrau district, Karaganda region, RK), which is known to be brucellosis free. Cows were vaccinated through the s.c. route in 2015 at the age of 5-6 months with a full dose of *B. abortus* 19 (8×10^10^ cells [Shchelkovo Biokombinat, Moscow region, Russia]); they were then subjected to reimmunization with the same vaccine at a dose of 8×10^9^ cells by administration to the conjunctiva during the 3 years that followed. Twelve sera obtained from unvaccinated cattle that were seronegative as determined by RBT, AT, and CFT were used as control samples.

### Determination of the antigenicity of the recombinant proteins by ELISA

Briefly, wells of a polystyrene plate were coated with rOmp19, rOmp25, or rOmp31 as described above. Rabbit antiserum to *B. abortus* 19 WC or cattle serum were diluted in 8 recombinant protein-coated wells, starting with 1:100 in PBS-T; the plate was maintained at 37°C for 1 h. Antibodies bound to the plate were detected with HRP-conjugated goat anti-rabbit (Jackson ImmunoResearch, West Grove, USA) or rabbit anti-bovine antibodies (Sigma-Aldrich, St. Louis, USA). To determine the titer of the rabbit antibodies, the dilution of the antiserum was taken, the OD of which was two or more times higher than the OD of the negative control serum at a dilution of 1:100. A cutoff value for serodiagnosis in cattle was calculated using the mean OD of 12 *B. abortus*-negative control sera; the results were considered to be positive if the OD value of the well with test serum (ODt) was at least two-fold higher than that of the control well (ODc) with both sera at a dilution of 1:100.

### Procedure of RBT

RBT was carried out according to the manufacturer’s instructions (Antigen, Almaty, Kazakhstan).

### Gel electrophoresis and Western blot analyses

The rOmps were individually fractionated on 12% sodium dodecyl sulfate-polyacrylamide gel [24] and then transferred onto a 0.45 μm pore-size nitrocellulose membrane (Watman Nytran Supercharge Aldrich, St. Louis, USA); immunoblotting was performed using standard methods [25]. After washing and blocking, membrane was incubated with diluted serum (1:100) from a cow (N6) that was experimentally infected with *B. abortus* 544 and sampled on day 28 p.i. or with rabbit antiserum to *B. abortus* 19 WC. HRP-conjugated secondary antibodies were used at a dilution of 1:2000. Immunoreactive bands were detected after developing with 4-chloro-1-napthol (Sigma-Aldrich, St. Louis, USA).

### Statistical analysis

Statistical comparisons between the ODt/ODc mean values were performed using ANOVA. Statistical analysis of antibody titers was carried out according to a method that was previously described [26]. The results were considered significant at p<0.05.

## Results

**Figure-1:**
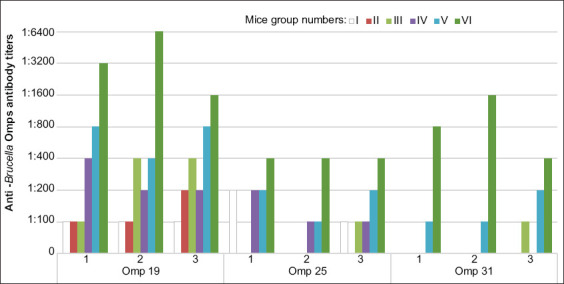
Immunogenicity of *Brucella* Omp19 in mice by enzyme-linked immunosorbent assay.

Mice in Group II were determined to have generated antibodies against *Brucella* Omp19 after a single s.c. immunization even without the use of adjuvant (Figure-1).

The immunization schedules used to generate antibodies in mice in Groups III, IV, and V have resulted in an overall increase in antibody production; however, positive results in the ELISA for rOmp25 and rOmp31 were also observed in response to the lowest dilutions of the serum samples from most of the mice immunized with rOmp19 emulsified in FCA or FIA. It is necessary to emphasize that sera from mice injected with FCA only (Group I) also revealed positive results and were capable of detecting both rOmp19 and rOmp25 when use at a 1:100 or 1:200 dilution. High titer of anti-rOmp19 antibodies (1:1600-1:6400) was detected in serum samples from mice in Group VI after triple immunization with Freund’s adjuvants. However, the resulting antisera also showed some cross-reactivity with rOmp25 and rOmp31.

The antigenicity of Omp19 was studied using sera from (i) rabbits immunized with killed *B. abortus* 19 WC, (ii) cattle that were experimentally infected with *B. abortus* 544, (iii) cows revaccinated against brucellosis, (iv) non-immunized cattle from new epizootic foci with positive RBT and/or CFT, and (v) unvaccinated seronegative heifers from a brucellosis-free farm. As shown in Table-1, Omp19 was noticeably inferior to the other proteins with respect to antigenicity specifically as indicated by the rabbit antisera.

**Table-1:** Antigenicity of the Omps in anti-*B. abortus* 19WC rabbit sera.

Individual numbers of rabbits	Days after immunization	Antibody titers against *Brucella* Omps by ELISA		

		Omp19	Omp25	Omp31
1	14	1:400	1:1600	1:800
2		1:800	1:1600	1:1600
1	21	1:1600	1:3200	1:3200
2		1:1600	1:3200	1:3200
1	35	1:3200	1:6400	1:3200
2		1:3200	1:6400	1:3200
1	45	1:6400	1:6400	1:6400
2		1:3200	1:6400	1:6400
Average antibody titers		1:980 (77.8%; −43.8%)	1:1970 (65.9%; −39.7%)	1:1490 (70.5%; −41.3%)

*B. abortus=Brucella abortus,* ELISA=Enzyme-linked immunosorbent assay, WC=Whole cell

As shown in Table-1, the average anti-Omp19 titers in antisera from rabbits sampled on days 14, 21, 35, and 45 after vaccination were significantly lower than those targeting Omp25 and Omp31 (p<0.05). However, these data indicate that Omp19, similar to Omp25 and Omp31, can be detected by rabbit anti-*B. abortus* 19 WC serum; these findings also confirm expression of the recombinant protein in *E. coli* BL21 in active state.

The findings in Table-2 reveal the antigenicity of Omp19 compared to that of Omp25 and Omp31 as shown in sera from cattle that were experimentally infected with *B. abortus* 544.

**Table-2:** Antigenicity of *Brucella* Omps as detected in sera from *B. abortus* 544-infected cattle.

Individual numbers of cows	Recombinant proteins used in ELISA as antigens					

	Omp19		Omp25		Omp31	

	Days p.i. with *B. abortus* 544					

	14	28	14	28	14	28

	The ratio of ODt to ODc					
6	3.6	11.5	5.5	14.2	1.7*	5.8
7	2.6	11.2	0.6*	3.5	1.0*	2.2
8	3.0	12.1	1.7*	8.1	0.8*	1.3*
9	2.9	10.9	2.3	9.6	1.8*	2.2
10	11.0	9.1	4.1	7.1	0.5*	2.4
16	5.4	12.0	2.7	9.0	3.6	2.5
17	4.6	11.8	1.5*	9.1	3.9	3.6
18	19.0	13.2	6.8	11.7	6.6	5.9
19	14.5	12.7	2.1	2.5	4.3	3.5
20	6.5	12.4	3.3	14.7	0.7*	0.8*
27	4.2	10.9	1.2*	8.4	1.6*	1.8*
30	5.5	11.6	3.2	8.1	4.0	2.2
ODt/ODc mean values	6.9±3.0	11.6±1.1	2.9±1.8	8.8±3.6	2.5±1.9	2.9±1.6

* Negative result. *B. abortus=Brucella abortus*, ELISA=Enzyme-linked immunosorbent assay

The ELISA designed to detect antibodies to Omp19 was determined to be more sensitive than the immunoassays used to detect the other recombinant proteins. For example, on day 14 p.i., antibodies against Omp19 were detected by ELISA in serum samples from all infected animals, while antibodies targeting Omp31 and Omp25 were detected in sera from 42% to 67% of the infected animals, respectively. Antibodies against Omp31 were not detected in three of the animals (N8, 20, and 27) either on days 14 and 28 p.i. Moreover, based on the values of ODt/ODc, there was no significant change in the level of anti-Omp31 antibody production during this period; by contrast, the specific humoral response to Omp19 and Omp25 became more prominent, at 6.9±3.0-11.6±1.1 (p<0.05) and 2.9±1.8-8.8±3.6 (p<0.01), respectively.

Antibody titers on day 14 p.i. were detected at levels that were indistinguishable from one another, including Omp19 at 1:650(+36.6%, −27.3%), Omp25 at 1:400 (+36.6%, −27.3%), and Omp31 at 1:420 (+18.9%, −15.9%). However, on day 28 p.i., the titer of antibodies targeting Omp19 was significantly higher (1:6400) than those targeting Omp25 or Omp31 (1:2600 and 1:2200, respectively; p<0.05).

**Figure-2:**
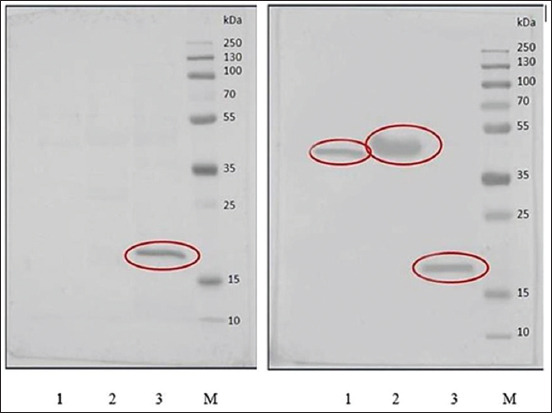
Western blot of Omps with cattle (left) and rabbit (right) antisera to *Brucella abortus*. Lane 1: Omp25; Lane 2: Omp31; Lane 3: Omp19; Lane 4: Molecular markers.

Results of immunoblotting are presented in Figure-2. Serum sampled on day 28 p.i. from cow N6 that had been infected with *B. abortus* 544 revealed only the Omp19 protein band; interestingly, antibodies from rabbits vaccinated with inactivated *B. abortus* 19 WC detect all three protein bands, including rOmp25, rOmp31, and rOmp19 with apparent MWs of 42, 48, and 19 kDa, respectively. These sizes agreed with the MWs determined for the rOmp25 and rOmp31 fusion proteins, respectively.

**Figure-3:**
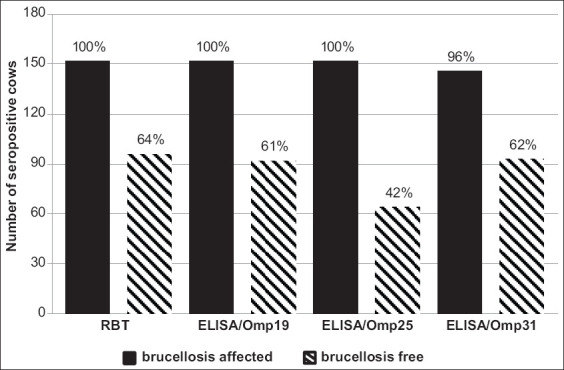
The reactivity of *Brucella* proteins in sera of cattle from farms with different epizootic status of brucellosis.

The antigenicity of the Omps as indicated in serum samples from cows maintained in either brucellosis-free or affected farms is indicated in Figure-3. In the serum samples from cows that had been revaccinated against *B. abortus* 19, antibodies were detected 10 months after the final immunization in more than half of the livestock (61-64%) by RBT, ELISA targeting Omp31 and ELISA targeting Omp19 with average titers of 1:210 (+3.5; −3.3%) and 1:280 (+4.2 %; −4.0%), respectively. Omp25 was found to be less antigenic in this setting; anti-Omp25 antibodies were detected in 42% of the cattle with an average titer of 1:250 (+5.0%; −4.8%). We emphasize that serum samples from nearly one-third of the cows (28%) contained no anti-Omp antibodies whatsoever. Antibodies to one or two of the three Omps were detected in 13% or 28% of these cows, respectively. A direct strong concordance with a correlation coefficient of 0.70 (p<0.01) was established between the results of ELISA targeting Omp19 and the ELISA targeting Omp31. A moderate correlation was observed between the ELISA targeting Omp19 and the ELISA targeting Omp25 (r=0.38; p<0.05) as well as between the ELISA targeting Omp31 and the ELISA targeting Omp25 (r=0.35; p<0.05).

**Table:** 

Serological tests				Serological tests			
	
RBT	ELISA targeting recombinant proteins			RBT	ELISA targeting recombinant proteins		
	
	Omp19	Omp25	Omp31		Omp19	Omp25	Omp31

Number of animals with positive results, head (%)				Number of animals with positive results, head (%)			
	152	152	146		92	64	93
152	Antibody titers			96	Antibody titers		
	1:280(+2,1; -2,1)	1:370 (+2,1; -2,1)	1:350 (+2,8; -2,7)		1:280 (+4,2; -4,0)	1:250 (+5,0; -4,8)	1:210 (+3,5; -3,3)

Agglutinating antibodies identified by RBT were identified in all seropositive cows from brucellosis-affected farms. The results of this test were fully confirmed by the results of the ELISA targeting Omp19 and the ELISA targeting Omp25 with average titers of 1:280 and 1:370 (+2.1%; −2.1%), respectively. Only 4% of the RBT seropositive animals had no anti-Omp31 specific antibodies. The average titer of antibodies to Omp31 was 1:350 (+2.8%; −2.7%). Comparing the mean antibody titers of cows from farms with different statuses with respect to brucellosis, there were no differences with respect to results from the ELISA targeting Omp19; however, this indicator was significantly higher in serum samples from cattle exposed to new epizootic foci when Omp25 and/or Omp 31 were evaluated as antigens (p<0.05).

## Discussion

Timely and reliable diagnosis of brucellosis is a key link in the fight against this widespread zoonotic infection. The search for a suitable antigen for serological diagnosis of brucellosis remains among the most urgent issues in current veterinary science. Over the past 30 years, many attempts have been made to identify immunologically active *Brucella* proteins. Advances in genetic engineering have made it possible to obtain recombinant proteins and to test their value in serological methods used to diagnose brucellosis. Omp25 [27], Omp31 [28], and Omp19 [29] are the first *Brucella* proteins to be cloned and expressed in recombinant form in *E. coli*. These recombinant proteins are also undergoing evaluation in the hopes of using them to create novel vaccines [30,31]. The potential of these proteins with respect to serologic diagnosis has been explored minimally, and the results obtained thus far are not always consistent with one another. For example, in one initial research study, none of the eight recombinant proteins used, including five Omps (Omp10, Omp16, Omp19, Omp25, and Omp36), were detected in sera from experimentally infected non-pregnant cows and sheep or from naturally infected cattle [13]. The antigenicity of recombinant Omp31 was studied by ELISA using human sera as well as those from animals infected with brucellosis; specific antibodies were identified in 48% of the human subjects, 61% of the infected sheep, and 87% of the infected dogs [32].

Many researchers believe that the potential usefulness of Omp antigens in combination with other recombinant proteins from *Brucella* should not be dismissed. Among these, Navarro-Soto *et al*. [33] generated rOmp31 from *Brucella ovis* through expression in *E. coli* and used the recombinant protein to demonstrate its high antigenicity using sera from *Brucella*-positive cattle. A serological diagnostic study using *B. melitensis* Omp31 in a study of goat and sheep brucellosis revealed that this target was associated with lower sensitivity but higher specificity on ELISA compared to RBT [14]. In the present work, we evaluated the antigenicity of Omp19 compared to that observed in response to Omp25 and Omp31. Our results revealed that Omp19 is a moderately immunogenic protein that can induce antibody production without an adjuvant. Furthermore, a single injection of the immunogen mixed with either FIA or FCA resulted in stimulation of the immune system and an associated increase in antibody titer to 1:800. However, these antibodies were cross-reactive with Omp25 and Omp31 at the lowest serum dilutions. Hyperimmune anti-Omp19 serum antibodies with a maximum titer of 1:6400 were obtained on day 28 p.i. using a triple immunization strategy and mixture adjuvants; however, these sera were also cross-reactive with Omp25 and Omp31 up to dilutions of 1:400 and 1:1600, respectively. These data suggest that the structures of the Omps may share similar epitopes. On the other hand, day 21 serum samples from mice injected with FCA reacted positively with both Omp19 and Omp25 at the lowest dilutions; this may be associated with the production of antibodies against adjuvant components that include mycobacterial cell wall and against peptidoglycan epitopes that are similar to those of *Brucella* spp. However, taken together, we conclude that Omp19 possesses humoral immunogenicity and can induce the production of specific antibodies after a single injection without the use of adjuvant.

Recombinant Omp19 was synthesized in *E. coli* in immunologically active form and was detected by rabbit antibodies that were generated against phenol-killed *B. abortus* 19 WC. The protein antigenicity was determined to be more pronounced in serological testing of cattle that were experimentally infected with *B. abortus* 544. Anti-Omp19 antibodies were detected in all infected animals on day 14 p.i., while the overall positive results from the ELISA targeting Omp25 were observed on day 28 p.i; antibodies to Omp31 were not detected in 25% of experimental animals at this time point. Moreover, on day 28 p.i., the anti-Omp19 antibody titer was significantly higher than the titers against the other two proteins. It is interesting to note that Omp19 was less reactive in anti-*B. abortus* 19 WC rabbit sera compared to Omp25 and Omp31, but it showed comparatively high antigenicity in serological testing of cattle infected with *B. abortus* 544 by ELISA. Apparently, Omp19 exposure may be different in live versus inactivated bacteria; as such, the results obtained from rabbits immunized with killed *Brucella* might be different from those obtained from experimentally infected cattle. We have previously described the strong antigenic properties of a 19 kDa protein detected on Western blots of soluble protein preparations from *B. abortus* and *B. melitensis* probed with serum from a cow that was naturally infected with brucellosis [34]. These data suggest that Omp19 may be more antigenic than the other *Brucella* Group 3 antigens. These results are consistent with the findings of Letesson *et al*. [13] who reported that antibody responses to the minor Omps, including Omp19, were somewhat greater than responses to the major Omps. The authors note that this may be associated with improved preservation of antigenicity. Omp19 is not an integral membrane protein but a surface lipoprotein [35]; as such, it is less likely that the membrane environment will have a profound impact on its antigenicity.

Rabbit serum antibodies obtained against inactivated *B. abortus* 19 WC bind to all these Omps evaluated by Western blot. By contrast, the protein bands corresponding to Omp25 and Omp31 were not detected in the blots probed with sera from experimentally infected cows. It is well known that many molecules become more immunogenic in response to denaturation. Phenol inactivation of *Brucella* WC preparation might result in a change in the structure of the Omps and could conceivably expose new epitopes. Denatured antigens may induce an antibody response against determinants that are not found on the native antigen [36]. We note that in an infected animal, the serologically critical epitopes of Omp25 and Omp31 are apparently less accessible to the immune system than are similarly critical determinants of Omp19. It is also possible that the denaturing conditions associated with the immunoblotting procedure might have an impact on the tertiary structure of the recombinant proteins. Finally, the antigenic and/or immunogenic properties of proteins might be different when comparing *in vitro* to *in vivo* conditions [7].

Omp19, similar to Omp25, exhibited high antigenicity in cows experiencing new brucellosis epizootic foci. Antibodies specific to Omps were also identified in more than half of the animals maintained in a brucellosis-free herd for as long as 10 months after revaccination with *B. abortus* 19. As such, we can conclude that antibodies to *Brucella* Omps are produced not only by infected animals but also by animals that have been vaccinated. The use of ELISAs that detect Omp19, Omp25, and Omp31 separately and ultimately reduced the test sensitivity; this might be related to the fact that a single protein might not be detected by all antibodies within the overall population.

## Conclusion

Taken together, our results suggest that recombinant *Brucella* Omp19 combined with Omp25 and/or Omp31 might be used as a reliable antigen to develop an ELISA for serological testing of cattle before initial vaccination. Further studies would be necessary to determine the efficacy of ELISAs targeting a combination of rOmps for the serological diagnosis of bovine brucellosis.

## Authors’ Contributions

AB designed the study. BI obtained and purified recombinant proteins. OA, AS, and ZhS performed the experiment and collected the serum samples. All authors drafted, read, and approved the final manuscript.

## References

[1] Franc K.A, Krecek R.C, Häsler B.N, Arenas-Gamboa A.M (2018). Brucellosis remains a neglected disease in the developing world:A call for interdisciplinary action. BMC Public Health.

[2] Nicoletti P (2010). Brucellosis:Past, present and future. Prilozi.

[3] Pappas G, Papadimitriou P, Akritidis N, Christou L, Tsianos E.V (2006). The new global map of human brucellosis. Lancet Infect. Dis.

[4] Bonfini B, Chiarenza G, Paci V, Sacchini F, Salini R, Vesco G, Villari S, Zilli K, Tittarelli M (2018). Cross reactivity in serological tests for brucellosis:A comparison of immune response of *Escherichia coli* O157:H7 and *Yersinia enterocolitica* O:9 vs *Brucella* spp. Vet. Ital.

[5] Moriyón I, Grilló M.J, Monreal D, González D, Marín C, López-Goñi I, Mainar-Jaime R.C, Moreno E, Blasco J.M (2004). Rough vaccines in animal brucellosis:Structural and genetic basis and present status. Vet. Res.

[6] Ko K.Y, Kim J.W, Her M, Kang S.I, Jung S.C, Cho D.H, Kim J.Y (2012). Immunogenic proteins of *Brucella abortus* to minimize cross reactions in brucellosis diagnosis. Vet. Microbiol.

[7] Navarro-Soto M, Morales-Loredo A, Álvarez-Ojeda G, Ramírez-Pfeiffer C, Tamez-Guerra P, Gomez-Flores R, Baddour M (2015). Recombinant proteins as antigens in serological diagnosis of brucellosis. Updates on Brucellosis.

[8] Cloeckaert A, Vizcaíno N, Paquet J, Bowden R, Elzer P (2002). Major outer membrane proteins of *Brucella* spp.:Past, present and future. Vet. Microbiol.

[9] Cloeckaert A, De Wergifosse P, Dubray G, Limet J (1990). Identification of seven surface-exposed *Brucella* outer membrane proteins by use of monoclonal antibodies:Immunogold labeling for electron microscopy and enzyme-linked immunosorbent assay. Infect. Immun.

[10] Tibor A, Decelle B, Letesson J.J (1999). Outer membrane proteins Omp10, Omp16, and Omp19 of *Brucella* spp. Are lipoproteins. Infect. Immun.

[11] Belzer C.A, Tabatabai L.B, Deyoe B (1991). Differentiation by Western blotting of immune responses of cattle vaccinated with *Brucella abortus* strain 19 or infected experimentally or naturally with virulent *Brucella abortus*. Vet. Microbiol.

[12] Hoffmann E.M, Shapiro S.J, Nicoletti P (1990). Evaluation of serologic and cellular immune responses of cattle to a nonlipopolysaccharide antigen from *Brucella abortus*. Am. J. Vet. Res.

[13] Letesson J.J, Tibor A, van Eynde G, Wansard V, Weynants V, Denoel P, Saman E (1997). Humoral immune responses of *Brucella*-infected cattle, sheep, and goats to eight purified recombinant *Brucella* proteins in an indirect enzyme-linked immunosorbent assay. Clin. Diagn. Lab. Immunol.

[14] Gupta V.K, Verma D.K, Singh S.V, Vihan V.S (2007). Serological diagnostic potential of recombinant outer membrane protein (Omp31) from *Brucella melitensis* in goat and sheep brucellosis. Small Rumin. Res.

[15] Lim J.J, Kim D.H, Lee J.J, Kim D.G, Min W, Lee H.J, Rhee M.H, Chang H.H, Kim S (2012). Evaluation of recombinant 28 kDa outer membrane protein of *Brucella abortus* for the clinical diagnosis of bovine brucellosis in Korea. J. Vet. Med. Sci.

[16] Manat Y, Shustov A.V, Evtehova E, Eskendirova S.Z (2016). Expression, purification and immunochemical characterization of recombinant OMP28 protein of *Brucella* species. Open Vet. J.

[17] Simborio H.L.T, Lee J.J, Reyes A.W.B, Hop H.T, Arayan L.T, Min W, Lee H.J, Yoo H.S, Kim S (2015). Evaluation of the combined use of the recombinant *Brucella abortus* Omp10, Omp19 and Omp28 proteins for the clinical diagnosis of bovine brucellosis. Microb. Pathog.

[18] Ahmed I, Khairani-Bejo S, Hassan L, Bahaman A, Omar A (2015). Serological diagnostic potential of recombinant outer membrane proteins (rOMPs) from *Brucella melitensis* in mouse model using indirect enzyme-linked immunosorbent assay. BMC Vet. Res.

[19] Bulashev A, Akibekov O, Suranshiyev Z.H, Ingirbay B, Eskendirova S (2019). Serodiagnostic potential of *Brucella* outer membrane and periplasmic proteins. Turk. J. Vet. Anim. Sci.

[20] Abkar M, Lotfi A.S, Amani J, Eskandari K, Ramandi M.F, Salimian J, Brujeni G, Alamian S, Kamali M, Koushki H (2015). Survey of Omp19 immunogenicity against *Brucella abortus* and *Brucella melitensis*:Influence of nanoparticulation versus traditional immunization. Vet. Res. Commun.

[21] Verma S, Rawat M, Kumawat S, Qureshi S, Mohd G, Tiwari A.K (2018). Protective role of *Brucella abortus* specific murine antibodies in inhibiting systemic proliferation of virulent strain 544 in mice and guinea pig. Vet. World.

[22] Bulashev A.K, Tursunov K.T, Kairova Z.K, Syzdykova A (2018). Obtaining the strain producing recombinant *Brucella abortus* Omp19 and studying its antigenicity. Bull. Kazatu.

[23] Bulashev A, Jakubowski T, Tursunov K, Kiyan V, Zhumalin A (2018). Immunogenicity and antigenicity of *Brucella* recombinant outer membrane proteins. Vet. Med. Zoot.

[24] Laemmli U.K (1970). Cleavage of structural protection during the assembly of the head of bacteriophage T4. Nature.

[25] Towbin H, Staehelin T, Gordon J (1979). Electrophoretic transfer of proteins from polyacrylamide gels to nitrocellulose sheets:Procedure and some applications. Proc. Natl. Acad. Sci. U. S. A.

[26] Saiduldin T.S (1981). Statistical analysis of the results of serological tests. Veterinariya.

[27] de Wergifosse P, Lintermans P, Limet J.N, Cloeckaert A (1995). Cloning and nucleotide sequence of the gene coding for the major 25-kilodalton outer membrane protein of * Brucella abortus*. J. Bacteriol.

[28] Vizcaino N, Cloeckaert A, Zygmunt M.S, Dubray G (1996). Cloning, nucleotide sequence, and expression of the *Brucella melitensis* Omp31 gene coding for an immunogenic major outer membrane protein. Infect. Immun.

[29] Kovach M.E, Elzer P.H, Robertson G.T, Chirhart-Gilleland R.L, Christensen M.A, Peterson K.M, Roop R.M (1997). Cloning and nucleotide sequence analysis of a *Brucella abortus* gene encoding an 18 kD immunoreactive protein. Microb. Pathog.

[30] Mailybayeva A, Yespembetov B, Ryskeldinova S, Zinina N, Sansyzbay A, Renukaradhya G.J, Petrovsky N, Tabynov K (2017). Improved influenza viral vector based *Brucella abortus* vaccine induces robust B and T-cell responses and protection against *Brucella melitensis* infection in pregnant sheep and goats. PLoS One.

[31] Paul S, Peddayelachagiri B.V, Nagaraj S, Konduru B, Batra H.V (2018). Protective and therapeutic efficacy study of divalent fusion protein rL7/L12-Omp25 against *B. abortus* 544 in presence of IFN?. Appl. Microbiol. Biotechnol.

[32] Cassataro J, Pasquevich K, Bruno L, Wallach J.C, Carlos A.F, Pablo C.B (2004). Antibody reactivity to Omp31 from *Brucella melitensis* in human and animal infections by smooth and rough. Brucella Clin Diagn. Lab. Immunol.

[33] Navarro-Soto M.C, Gomez-Flores R, Morales-Loredo A, Ramírez-Pfeiffer C, Tamez-Guerra P, Álvarez-Ojeda G (2014). Effective use of Recombinant *Brucella ovis* Omp31 Antigen to Detect Cattle Serum Antibodies by the ELISA Indirect Test. Biotechnology Summit, Santa María Huatulco, Oaxaca, Mexico.

[34] Bulashev A.K, Suranshiev Z.A, Zhumalin A.K, Tursunov K.A (2016). The antigenicity of the outer membrane proteins of *Brucella*. Biotechnol. Theory Pract.

[35] Tibor A, Saman E, de Wergifosse P, Cloeckaert A, Limet J.N, Letesson J.J (1996). Molecular characterization, occurrence, and immunogenicity in infected sheep and cattle of two minor outer membrane proteins of *Brucella abortus*. Infect Immun.

[36] Greenfield E.A, DeCaprio J, Brahmandam M (2018). Making weak antigens strong:Modifying protein antigens by denaturation. Cold Spring Harb. Protoc.

